# Compendium of Human Histology

**Published:** 1861-09

**Authors:** 


ART. VIII.—*Compendium of Human Histology.* By C. MOREL, Professor Agrégé a la Faculté de Médecine de Strasbourg. Illustrated by Twenty-eight Plates. Translated and Edited by W. H. VAN BUREN, M.D., Professor, etc. 8vo., pp. 207. New York: Bailliere Brothers, 1861.

THE work of M. Morel does not profess to be an elaborate treatise on Human Histology; it belongs rather to that very valuable and commendable class of publications which give the student an insight into the more profound portions of the subjects which they discuss, without allowing him to be overwhelmed by incomprehensible theories or conflicting views of observers. Such books are introductions to more extensive works on the same subjects, and were it not for their kindly guidance, many a confused mind would give up in despair the study of some of the most interesting facts in medical science.

The volume before us is, of course, descriptive anatomy, applied to minute points of structure, and, as in works on special anatomy, constant reference is made to the engravings, which constitute so important a feature of the book.

The letter-press of this Compendium of Histology would scarcely cover a hundred pages of the usual octavo volumes, for the blank margins are nearly as wide as the columns of printed matter. The engravings are very prettily executed, and the type is unusually large.

We agree with Dr. Van Buren, the editor, that the style of the author is precise and compendious. We think, however, that brief and comprehensive as the work is, it errs in assuming that "the reader is a good descriptive anatomist, and possesses a fair knowledge of general structure."

The notes of the editor and translator are almost wholly bibliographical or personally historical, and in this respect differ from the usual style of foot-notes, so often added to medical works for the self-glorification of the editor, who, not content with seeing his name in print on the title-page, endeavors to improve on the views of his author by gratuitous insertions of his own.

When mention is made (p. 43) that two translations of Kölliker's admirable treatise on "Human Microscopic Anatomy" have appeared, it should also be stated that in 1859 the Sydenham Society's translation was republished in Philadelphia, with excellent annotations by Dr. Jacob Da Costa.

